# Comparison of gross target volumes based on four‐dimensional CT, positron emission tomography‐computed tomography, and magnetic resonance imaging in thoracic esophageal cancer

**DOI:** 10.1002/cam4.3072

**Published:** 2020-06-08

**Authors:** Huimin Li, Fengxiang Li, Jianbin Li, Youzhe Zhu, Yingjie Zhang, Yanluan Guo, Min Xu, Qian Shao, Xijun Liu

**Affiliations:** ^1^ Weifang Medical University Weifang China; ^2^ Department of Radiation Oncology Shandong Cancer Hospital and Institute Shandong First Medical University and Shandong Academy of Medical Sciences Jinan China; ^3^ Cheeloo College of Medicine Shandong University Jinan China; ^4^ School of Medicine and Life Sciences University of Jinan, Shandong Academy of Medical Sciences Jinan China; ^5^ Department of PET‐CT Shandong Cancer Hospital and Institute Shandong First Medical University and Shandong Academy of Medical Sciences Jinan China

**Keywords:** esophageal cancer, four‐dimensional computer tomography, gross target volume, magnetic resonance imaging, positron emission tomography

## Abstract

**Purpose:**

The application value of ^18^F‐FDG PET‐CT combined with MRI in the radiotherapy of esophageal carcinoma was discussed by comparing the differences in position, volume, and the length of GTVs delineated on the end‐expiration (EE) phase of 4DCT, ^18^F‐FDG PET‐CT, and T_2_W‐MRI.

**Methods:**

A total of 26 patients with thoracic esophageal cancer sequentially performed 3DCT, 4DCT, ^18^F‐FDG PET‐CT, and MRI simulation for thoracic localization. All images were fused with the 3DCT images by deformable registration. GTV_CT_ and GTV_50%_ were delineated on 3DCT and the EE phase of 4DCT images, respectively. The GTV based on PET‐CT images was determined by thresholds of SUV ≥ 2.5 and designated as GTV_PET2.5_. The images of T_2_‐weighted sequence and diffusion‐weighted sequence were referred as GTV_MRI_ and GTV_DWI_, respectively. The length of the abnormality seen on the 4DCT, PET‐CT, and DWI was compared.

**Results:**

GTV_PET2.5_ was significantly larger than GTV_50%_ and GTV_MRI_ (*P* = .000 and 0.008, respectively), and the volume of GTV_MRI_ was similar to that of GTV_50%_ (*P* = .439). Significant differences were observed between the CI of GTV_MRI_ to GTV_50%_ and GTV_PET2.5_ to GTV_50%_ (*P* = .004). The CI of GTV_MRI_ to GTV_CT_ and GTV_PET2.5_ to GTV_CT_ were statistically significant (*P* = .039). The CI of GTV_MRI_ to GTV_PET2.5_ was significantly lower than that of GTV_MRI_ to GTV_50%_, GTV_MRI_ to GTV_CT_, GTV_PET2.5_ to GTV_50%_, and GTV_PET2.5_ to GTV_CT_ (*P* = .000‐0.021). Tumor length measurements by endoscopy were similar to the tumor length as measured by PET and DWI scan (*P* > .05), and there was no significant difference between the longitudinal length of GTV_PET2.5_ and GTV_DWI_ (*P* = .072).

**Conclusion:**

The volumes of GTV_MRI_ and GTV_50%_ were similar. However, GTV_MRI_ has different volumes and poor spatial matching compared with GTV_PET2.5_.The MRI imaging could not include entire respiration. It may be a good choice to guide target delineation and construction of esophageal carcinoma by combining 4DCT with MRI imaging. Utilization of DWI in treatment planning for esophageal cancer may provide further information to assist with target delineation. Further studies are needed to determine if this technology will translate into meaningful differences in clinical outcome.

## Graphical abstract

Target volume delineation guided by multimodality images has become the key technology for precise radiotherapy of esophageal cancer. It may be a good choice to guide target delineation and construction of esophageal carcinoma by combining 4DCT with MRI imaging. There are significant differences for the GTVs in size and spatial position derived from T2W‐MRI and FDG‐PET/CT. Whereas, DW‐MRI could be used to replace PET‐CT for determining the upper and lower boundaries of esophageal carcinoma.

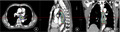

## 1 INTRODUCTION

Esophageal cancer (EC) is regarded as one of the most aggressive malignancies, which ranked seventh in incidence and sixth in cancer‐related death worldwide. It is estimated that there will be approximately 258 000 new cases and over 193 000 deaths for EC in 2018.^1^ For patients with medically inoperable tumors, definitive chemoradiation (dCRT) is preferred. For patients with locally advanced esophageal cancer, standard therapy with curative intent consists of neoadjuvant chemoradiation therapy followed by surgery, with 5‐year survival improving by 20%.^2^ However, local‐regional persistence and relapse of disease account for the majority of radiation treatment failures, with local relapse rate of 44%.^3^, ^4^ The majority of local failures occur within the gross tumor volume (GTV). Hence, it has become increasingly important to delineate the GTV precisely. Recently, advances in multimodality imaging have made a profound impact on the definition of target volumes of esophageal cancer, which could improve target coverage with a much steeper dose gradient and reduce irradiated normal tissues.

Currently, delineation of esophageal tumors is performed on computed tomography (CT), and the added ^18^F‐fluorodeoxyglucose (FDG) positron emission tomography (PET) have been explored.^5^ It is well‐known that conventional three‐dimensional CT (3DCT) images were acquired during free breathing. Motion derived from respiration and heart beating adds to the challenges to precise delineation of target volumes on 3DCT. The artifacts caused by respiratory motion can be reduced by the implementation of four‐dimensional CT (4DCT) techniques, and the most stable sequence for the end‐expiration (EE) phase of 4DCT scans were frequently selected for target delineation.^6^, ^7^, ^8^ However, delineation on 4DCT only is challenging, mainlly in poorly differentiating tumor from nomal tissue at the mediastinal tumor borders. The use of ^18^F‐FDG PET‐CT images could distinguish the tumor from normal tissues and reduce the interobserver variability by automated contour, but it is difficult to widely and repeatedly use in clinical practice limited by its poor spatial resolution, high price, and radiation injury.^10^

Magnetic resonance imaging (MRI) is non‐invasive, non‐radiating, and provides an excellent soft‐tissue contrast. With the advance of MRI‐guided radiation delivery, MRI has been increasingly recommended and incorporated into treatment guidelines in EC.^11^, ^12^, ^13^ Especially, on T_2_ weighted (T_2_W) turbo spin echo (TSE) sequence, MRI can well show the contour of the tumor based on the thickening of the wall and the signal change of this lesion,^4^ suggesting that the use of MRI could be more accurate than CT to delineate GTVs of esophagus. Previously, it has been shown that the use of 4DCT combined with PET could improve the exact of target region.^8^ As the wide application of MRI simulation, the value of MRI image in target volume delineation during multimodality imaging has been focused on, especially, the correlation in GTVs contouring for esophageal tumors between some particular sequence of MRI with PET‐CT and 4DCT imaging. In this study, we compared the differences in position, volume, and the length of GTVs derived from MRI, and PET‐CT images, directly or indirectly, based on the medium of 3DCT and end‐expiration of 4DCT. Our purpose is to explore the necessity of combing MRI with 4DCTor PET‐CT imaging in delineating GTVs of esophageal cancer.

## 2 MATERIALS AND METHODS

### 2.1 Patients selection and characteristics

After receipt of the ethics board of our hospital approval, a total of 31 patients with pathologically confirmed thoracic esophageal cancersequentially performed 3DCT, 4DCT, ^18^F‐FDG PET‐CT, and MRI simulation between November 2016 and August 2017. Inclusion criteria for this study were: (a) the patients with histologically proven thoracic esophageal carcinoma; (b) the patients who were medically unsuitable for or declined surgical treatment; (c) the patients with no contraindications to chemoradiotherapy; (d) the patients who have no previous thoracic radiotherapy and no history of thoracic malignance; (e) the patients who have basically normal cardiopulmonary function; (f) the patients who signed the informed consent. According to local multidisciplinary team (MDT) decision, concurrent chemoradiotherapy is generally recommended for patients. All patients voluntarily underwent 3DCT, 4DCT, ^18^F‐FDG PET‐CT, and MRI simulation scanning and were given provision of fully informed consent before participation. The exclusion criteria were as follows: (a) patients with maximal standardized uptake value (SUV) on PET of less than 2.0 (n = 1); (b) patients with poor quality of simulation MR images (n = 2); (c) patients with metastatic lymph nodes closely adjacent to primary tumor on PET‐CT (n = 2). Consequently, the image data from 26 patients were available for analysis. Patients characteristics are displayed in Table 1.

**TABLE 1:** Characteristics of the patients enrolled in the study

Patients	Sex	Age, y	Tumor location^a^	SUV_max_	Pathology type	TNM stage^b^
1	Male	62	Middle	22.6	Squamous	T3N3M0
2	Male	59	Upper	12.12	Squamous	T3N2M0
3	Male	67	Upper	7.67	Squamous	T3N2M0
4	Male	53	Middle	4.62	Squamous	T2N2M0
5	Male	74	Upper	13.51	Squamous	T2N1M0
6	Female	71	Distal	7.82	Squamous	T2N1M0
7	Male	52	Upper	16.02	Squamous	T3N2M0
8	Male	67	Middle	18.94	Squamous	T2N2M0
9	Male	71	Upper	22.33	Squamous	T3N2M0
10	Female	71	Upper	14.19	Squamous	T3N1M0
11	Female	72	Middle	15.66	Squamous	T2N2M0
12	Male	64	Distal	12.34	Squamous	T3N2M0
13	Male	65	Upper	11.71	Squamous	T3N3M0
14	Male	71	Distal	22.86	Squamous	T3N2M0
15	Male	61	Middle	14.00	Squamous	T2N2M0
16	Male	66	Middle	5.80	Squamous	T3N2M0
17	Female	73	Distal	19.88	Squamous	T3N0M0
18	Male	72	Distal	17.41	Squamous	T2N2M0
19	Male	71	Middle	3.32	Squamous	T3N1M0
20	Male	47	Middle	14.45	Squamous	T2N2M0
21	Male	52	Middle	5.42	Squamous	T3N1M0
22	Male	62	Upper	12.02	Squamous	T3N3M0
23	Male	50	Upper	5.45	Squamous	T2N2M0
24	Female	71	Upper	12.32	Squamous	T3N1M0
25	Female	53	Distal	12.47	Squamous	T3N2M0
26	Male	67	Distal	7.79	Squamous	T2N1M0

Abbreviation: SUV_max_, maximum standardized uptake value.

^a^ American Joint Committee on Cancer (AJCC) classification 2017.

^b^ Clinical tumor‐node‐metastasis (cTNM) stage according to 8th edition TNM classification.

### 2.2 Image simulation and acquisition

During the simulation, all patients were immobilized using thermoplastic mask in the supine position with the arms raised above the head. Contrast enhanced (CE)‐3DCT and CE‐4DCT Images were obtained from the neck to the mid‐abdomen using the helical CT mode (ranging from the cricothyroid membrane down to the lower margin of the celiac trunk by 3DCT and ranging from the chest entrance to the lower margin of the cardia by 4DCT). All these scans were gathered during free breathing (FB) without any breathing control. For each person, an axial contrast‐enhanced 3DCT scan of the thoracic region was performed followed by a 4DCT scan under untrained free breathing conditions on a 16‐slice CT scanner (Philips Brilliance Bores CT). For 3DCT, each scan (360° rotation) took 1s to acquire followed by a 1.8 s dead time with a 2.4‐cm coverage. The 3DCT scanning procedure takes about 30s. During the 4DCT scanning, the respiratory signal was recorded with the Varian Real‐time Positioning Management (RPM) gating system by tracking the trajectory of infrared markers placed on the patients’ abdomen. Advantage 4D software sorts the reconstructed 4DCT images into ten respiratory phases, with 0% corresponding to end‐inhalation and 50% corresponding to end‐exhalation. Subsequently, the PET‐CT simulation images were scanned on the same day after CT scans. During free breathing (FB) without any breathing control, the patients lay on a flat table using thermoplastic mask with the laser alignment lines placed on the same position as the 3DCT, 4DCT infrared markers, to keep the same position with the 3DCT and 4DCT simulation scans. On the second day of PET‐CT simulation, MRI scans were conducted on a Philips Achieva 3.0T MRI scanner with body coil placed closely patients’ thorax to maximize signal, and patients positioned similarly supine on the scanning bed. MRI scanning consisted ofT2 weighted (T2w) turbo spin echo (TSE) and diffusion‐weighted (DW)MR images of b = 600 s/mm^2^. T2W‐MR images were obtained by respiratory‐triggered and pulse‐gating techniques, and scans were only acquired in end‐expiration.

### 2.3 Target delineation and image registration

**FIGURE 1:**
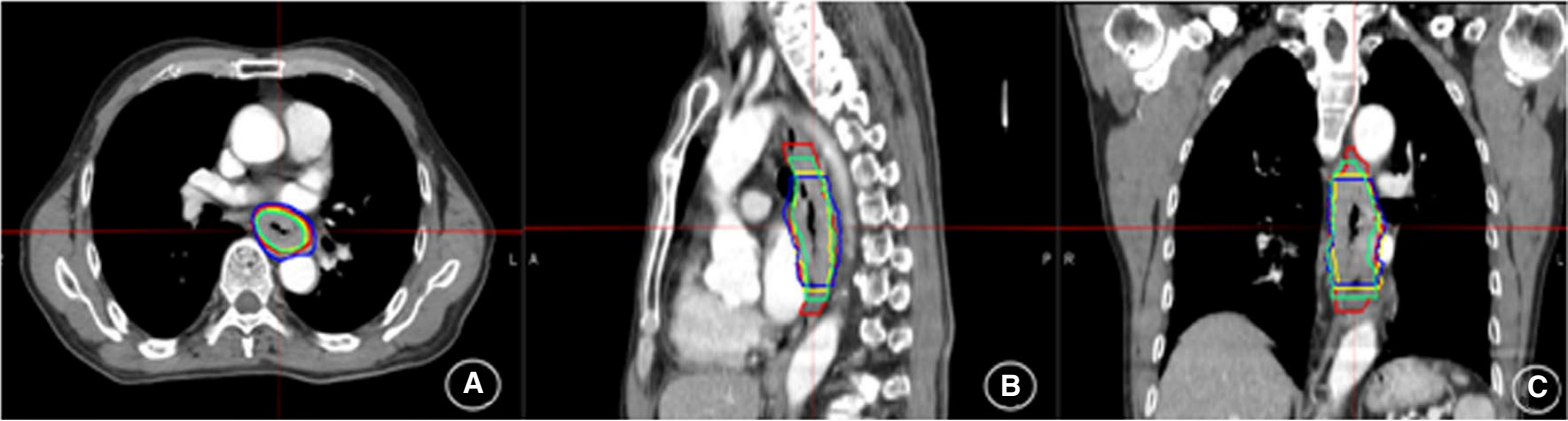
The picture of gross target volumes delineated on 3DCT(red), the EE phase of 4DCT(green), PET‐CT by the thresholds of SUV ≥ 2.5(blue), and T2W‐MRI(yellow) on transversal (A), sagittal (B), and coronal (C) in thoracic esophageal cancer

The 3DCT, 4DCT, PET‐CT, and MRI images were imported into the MIM version 6.7.6 software (Cleveland, USA).In order to ensure the accuracy and repeatability of the delineation of target volumes. By the same experienced radiation oncologist, GTV_S_ were manually delineated on 3DCT and the EE phases of 4DCT according to the same window setting (window width: 400HU and window level: 40 HU). Concurrently, the observers were instructed to delineate with the consensus guidelines, as the thickness of tube wall >5 mm (localized or circumscribed thickening of the esophageal wall and/or irregular narrowing of the local lumen) or the diameter without gas >10 mm.^14^ GTV_CT_ and GTV_50%_ were delineated on 3DCT and the EE phase of 4DCT images, respectively. The GTV based on PET‐CT images (GTV_PET_) was automatically contoured by the thresholds of SUV ≥2.5 and designated as GTV_PET2.5_. Actually, all the noncancerous regions within the GTV_PET_, including the areas overlaid by the heart, bone, and great vessels, were corrected to exclude manually with the help of the CT component of PET/CT. The images of T_2_W‐MRI and the fusion images of DW‐MRI and CT were referred as GTV_MRI_ and GTV_DWI_, respectively. During the registration process, 3DCT simulation was regarded as the main sequence. Meanwhile, the 4DCT, PET, and MRI images were used as the subordinated sequence. To reduce the effects of scanning mode and breathing movement in mediastinum, deformable image registration was undertaken using MIM software between 3DCT and other images (Figure 1).

### 2.4 Parameter evaluation

Both target volumes and longitudinal length of GTVs defined by 3DCT, 4DCT, PET‐CT, and MRI images were measured separately. Additionally, the differences of GTV_CT_, GTV_50%_, GTV_MRI_, and GTV_PET2.5_ in position were evaluated respectively, the degree of inclusion (DI) and the conformity index (CI) were calculated for the GTV_MRI_ and GTV_50%_, the GTV_PET2.5_ and GTV_50%_, GTV_MRI_ and GTV_CT_, the GTV_PET2.5_ and GTV_CT_ indirectly, and the GTV_MRI_ and GTV_PET2.5_directly. The definition of CI of volume A and B [CI (A, B)] was computed according to Struikmans et al.^15^ The formula was as follows:

$$\text{CI}\left(A,\;B\right)=\frac{A\cap B}{A\cup B}$$

The definition of DI of volume A included in volume B, [DI (A in B)] is the intersection between volume A and volume B divided by volume A.^16^The formula is as follows:

$$\text{DI}\left(\text{A in B}\right)=\frac{A\cap B}{A}$$

DW‐MR images of b‐values of 600 s/mm^2^ were used for GTV assessment group by defining high‐intensity regions^17^; At the axial level, the longitudinal lengths of the GTV were measured in terms of the number of DWI scanning layers. Analogously, the longitudinal lengths of the GTV of PET were calculated by thresholds of SUV ≥ 2.5 in terms of the number of PET scanning layers at the axial level. Tumor length measurements on endoscopy were observed by the endoscopic physician. Simultaneously, another senior endoscopic physician reviewed the length according to the same diagnostic criteria.

### 2.5 Statistical analysis

Statistical analysis was performed using the SPSS software package (SPSS 19.0). Descriptive statistics were used as appropriate. The Wilcoxon signed‐rank test was used to compare the target volumes that did not follow a normal distribution. The paired sample Student's *t* test was used for comparison of CI, DI, and the longitudinal lengths of lesion. Values of *P* < .05 were regarded as significant.

## 3 RESULTS

### 3.1 Volume analysis of GTVs

The target volumes defined using 3DCT,4DCT,PET‐CT, and MRI are listed in Table 2. The median volume variability between the GTV_PET2.5_ and GTV_50%_ and between the GTV_PET2.5_and GTV_MRI_ was statistically significant (*Z* = 4.458 and 2.654, *P* = .000 and 0.008, respectively), while there was no significant difference between GTV_PET2.5_ and GTV_CT_ (*P* > .05). Moreover the median volume variability between the GTV_MRI_ and GTV_CT_ was statistically significant (*Z *= −3.746,* P* = .000), while the volume of GTV_MRI_ was similar to that of GTV_50%_ (*P* > .05).

**TABLE 2:** Summary of volume of GTVs contoured using 3DCT, 4DCT, PET‐CT, and MRI

Modality	Target volumes(cm^3^)			Mean GTV Volume(Statistically Significant p‐values)
	Median	Range		
		Min	Max	
GTV_CT_	25.88	5.17	118.71	GTV_PET2.5_ < GTV_CT_, *P* = .069
GTV_50%_	22.57	4.35	109.88	GTV_PET2.5_ > GTV_50%_, *P* = .000;GTV_PET2.5_ > GTV_MRI_, *P* = .008
GTV_PET2.5_	24.70	4.99	121.10	GTV_MRI_ < GTV_CT_, *P* = .000
GTV_MRI_	23.18	4.43	104.88	GTV_MRI_ > GTV_50%_, *P* = .439

### 3.2 Positional analysis of GTVs

The CI comparing the various GTVs delineated on 3DCT, 4DCT, PET‐CT, and MRI are summarized in Table 3. Significant differences were observed between the mean CI of GTV_MRI_ to GTV_50%_ and GTV_PET2.5_ to GTV_50%_ (*P* < .05). Meanwhile, the mean CI between GTV_MRI_ and GTV_CT_ was significantly larger than that of GTV_PET2.5_ and GTV_CT_ (*P* < .05). The CI mean value for the GTV_MRI_ ‐GTV_PET2.5_ was 0.55 ± 0.09, which was significantly lower than that of GTV_MRI_ to GTV_50%_, GTV_MRI_ to GTV_CT_, GTV_PET2.5_ to GTV_50%_, and GTV_PET2.5_ to GTV_CT_ (*t *= −5.974,‐2.467,‐7.549,‐7.914, all *P* < .05, respectively, Table 3).

**TABLE 3:** CI of target volume defined using 3DCT, 4DCT, PET‐CT, and MRI (mean ± SD)

Target volume	CI	*t*‐value	*P*‐value
GTV_MRI_‐GTV_50%_	0.66 ± 0.08	−3.191	.004
GTV_PET2.5_‐GTV_50%_	0.59 ± 0.11		
GTV_MRI_‐GTV_CT_	0.68 ± 0.06	−2.185	.039
GTV_PET2.5_‐GTV_CT_	0.63 ± 0.11		
GTV_MRI_‐GTV_PET2.5_	0.55 ± 0.09		<.05^a^

Abbreviations: GTV, gross tumor volume; CI, the conformity index

^a^ The CI of GTV_MRI_ to GTV_PET2.5_was significantly lower than that of GTV_MRI_ to GTV_50%_, GTV_MRI_ to GTV_CT_, GTV_PET2.5_ to GTV_50%_ and GTV_PET2.5_ to GTV_CT_(*t *= −5.974, −2.467, −7.549, −7.914,*P* = .000, .021, .000, .000, respectively).

The DI of target volume defined using 3DCT, 4DCT, PET‐CT and MRI are included in Table 4. The DI of GTV_MRI_ in GTV_50%_ was significantly larger than that of GTV_PET2.5_in GTV_50%_ (*P* = .000). While there were no significant differences between the DI of GTV_50%_ in GTV_MRI_ and GTV_PET2.5_ (*P* = 1.101). The mean DI of GTV_MRI_ in GTV_CT_ was 0.87, which showed a significant difference than that of GTV_PET2.5_ in GTV_CT_ (*P* = .001).However, the DI of GTV_CT_ in GTV_MRI_ and GTV_CT_ in GTV _PET2.5_ show no significant difference (*P* = .707). In addition, The DI of GTV_PET2.5_ in GTV_MRI_ was significantly smaller than that of GTV_50%_ or GTV_CT_ in GTV_MRI_ (*P* = .000 and 0.000, respectively). Similarly, the DI of GTV_MRI_ in GTV_PET2.5_ was significantly smaller than that of GTV_50%_ or GTV_CT_ in GTV_PET2.5_ (*P* = .034 and .014, respectively).

**TABLE 4:** DI of target volume defined using 3DCT, 4DCT, PET‐CT, and MRI (mean ± SD)

Parameters	GTV_50%_ in GTV_MRI_	GTV_50%_ in GTV_PET2.5_	GTV_MRI_ in GTV_50%_	GTV_PET2.5_ in GTV_50%_	GTV_CT_ in GTV_MRI_	GTV_CT_ in GTV_PET2.5_	GTV_MRI_ in GTV_CT_	GTV_PET2.5_ in GTV_CT_	GTV_PET2.5_ in GTV_MRI_	GTV_MRI_ in GTV_PET2.5_
DI	0.79 ± 0.10	0.70 ± 0.10	0.79 ± 0.09	0.77 ± 0.09	0.87 ± 0.06	0.79 ± 0.10	0.76 ± 0.09	0.75 ± 0.09	0.74 ± 0.10	0.68 ± 0.10
*t*‐value	4.268		0.282		−3.887		−0.381			
*P*‐value	.000		1.101		.001		.707		<.05^a^	<.05^b^

Abbreviations: DI, the degree of inclusion; GTV, gross tumor volume.

^a^ The DI of GTV_PET2.5_ in GTV_MRI_ was significantly smaller than that of GTV_50%_or GTV_CT_ in GTV_MRI_ (*t *= −7.771, −4.151, *P* = .000, .000, respectively).

^b^ The DI of GTV_MRI_ in GTV_PET2.5_ was significantly smaller than that of GTV_50%_ or GTV_CT_ in GTV_PET2.5_ (*t *= −2.253, −2.645,*P* = .034, .014, respectively).

### 3.3 Longitudinal length measurements of GTVs

The comparison among tumor length measured by endoscopy, 3DCT, 4DCT, PET, and DWI are listed in Table 5. The tumor length measured by endoscopy is 4.48 ± 1.29 cm. The increasing order was tumor length measured by endoscopy, tumor length obtained by DWI scan, tumor length measured by PET scan, tumor length measured by 4DCT scan, and tumor length measured by 3DCT scan. The longitudinal length of GTV_CT_ was the longest, which showed a significant difference than that of GTV_50%_, GTV_PET2.5_, and GTV_DWI_ (t = 6.258,9.371,9.837, *P* = .000, .000, .000, respectively). The longitudinal length of GTV_50%_ was significantly larger than GTV_PET2.5_ and GTV_DWI_ (*t* = 8.625, 9.268, *P* = .000, .000, respectively). In addition, tumor length measurements by endoscopy were similar to the tumor length as measured by PET and DWI scan (*P* > .05), and there was no significant difference between the longitudinal length of GTV_PET2.5_ and GTV_DWI_ (*t* = 1.879, *P* = .072).

**TABLE 5:** Comparison among tumor length measured by endoscopy, 3DCT, 4DCT, PET, and DWI (mean ± SD)

Target	Length(cm)	Different length between four imagings and endoscopy(cm)	*t*‐value	*P*‐value
GTV_CT_	6.97 ± 1.73	2.43 ± 1.23	10.092	.000
GTV_50%_	6.84 ± 1.81	2.26 ± 1.24	9.315	.000
GTV_PET_	4.79 ± 1.58	0.12 ± 0.89	1.976	.061
GTV_DWI_	4.49 ± 1.33	−0.17 ± 0.77	−0.503	.620

## 4 DISCUSSION

Currently, target volume delineation guided by multimodality images has become the key technology for precise radiotherapy of esophageal cancer. Positron emission tomography‐magnetic resonance imaging (PET‐MRI) is a hybrid imaging technology that incorporates magnetic resonance imaging soft tissue morphological imaging and positron emission tomography functional imaging.^18^, ^19^ The use of PET‐MRI would have great value in improving radiation precision for esophageal cancer due to complementary imaging structures of PET and MRI. Comparative analysis of target volumes derived from 4DCT, PET‐CT, and MRI imaging would contribute to clinical application of multimodality images.

Based on our study, the results showed that the volume of GTV_MRI_ was similar to that of GTV_50%_. Moreover the median volume variability between the GTV_MRI_ and GTV_PET2.5_ and between the GTV_MRI_ and GTV_CT_ was statistically significant, while there was no significant difference between GTV_PET2.5_ and GTV_CT_, which were accorded with results reported in other literatures.^20^, ^21^ Guo et al's analysis^8^ demonstrated that GTV_PET2.5_ resembled better with IGTV_10_, which included GTVs contoured on ten phase of 4DCT. It revealed a trend that 3D PET image included some individualized information derived from respiratory motion. Furthermore, Karki et al^9^ reported that the size of GTVs on MRI were significantly smaller than those on 3DCT for lung cancer. In clinical practice, the MRI images were acquired during the respiratory cycle in the end‐exhale phase. Theoretically, the artifact included in MRI scans of the esophagus, which derived from the respiratory and cardiovascular movements, could have been reduced by respiratory‐triggered technique optimizations.^22^, ^23^ Our study proved the assumption. The results showed the volume of GTVs on T_2_W‐MRI was similar to that of GTVs contoured on EE phase of 4DCT, while the volume of GTV_MRI_ was much smaller than that of GTV_CT_ or GTV_PET_, respectively.

Although the size of GTVs derived from T_2_W‐MRI was similar to that from EE phase of 4DCT, the similarity and inclusion relation between the GTVs from the two images were unsatisfied. Our results indicated that there were poor CIs (0.66 ± 0.08) and mutual DIs (0.79 ± 0.09, 0.79 ± 0.10) between GTV_MRI_ and GTV_50%_, which suggested a great nonconformity for the two volumes. Given the data from our study, the motion information included in T_2_W‐MRI was closest to that in the end‐exhale phase of 4DCT, compared with conventional 3DCT and PET‐CT obtained throughout the free‐breathing cycle. Comparison between GTV_MRI_ and GTV_50%_ could reveal the intrinsic difference in distinguishing the boundary of esophageal carcinoma for MRI and CT imaging at best, due to reducing the influence of respiration motion at the most extent. Hence, the primary cause of spatial mismatch between GTV_MRI_ and GTV_50%_is the difference in showing the boundary of esophageal carcinoma for MRI and CT imaging. Most studies supported that T_2_W‐MRI could show the extent of esophageal tumor more clearly than CT.^4^ What's more, the CT imaging might overestimate the tumor length than MRI due to inflammatory edema of the esophageal wall after ischemic necrosis, with coincidence rate of 37.8% between CT scan and pathological specimens vs 76% between MRI scan and pathology.^4^, ^24^, ^25^ Therefore, MRI imaging may provide a valuable supplement to CT imaging in determining target volume for esophageal carcinoma, but we should be very cautious in using MRI imaging alone due to incomplete respiration information included in it. It may be a good choice to determine target volume for esophageal carcinoma that combines 4DCT with MRI imaging.

Vali et al^16^ confirmed that a threshold of approximately 2.5 yields the highest conformity index(CI) and best approximates the GTV_CT_. Moreover most studies noted that the interobserver variability, as well as the intraobserver variability, was significantly reduced when the ^18^F‐FDG PET image was available for tumor volume delineation.^26^, ^27^ So that more and more radiation oncologists believe that target volume delineation cannot be adequately performed without the use of ^18^F‐FDG PET. As always, radiation oncologists have been expecting to replace PET‐CT simulation by MRI for providing anatomical and functional imaging of esophageal carcinoma, due to exorbitant prices and radiation exposure of PET‐CT simulation. In this study, we evaluated the difference in matching and inclusion relation between the GTVs derived from simulation PET‐CT and MRI. Our results indicated that the mean CI of GTV_MRI_ to GTV_CT_ and GTV_MRI_ to GTV_50%_ were significantly larger than that of GTV_PET2.5_ to GTV_CT_ and GTV_PET2.5_ to GTV_50%_. Suggesting that there were greater mismatching between GTV_PET_ and GTV_CT_ or GTV_50%_, respectively. Moreover the lowest poor CIs (0.55) and mutual DIs (0.68, 0.74) also suggested great nonconformity between GTV_MRI_ and GTV_PET_. Perhaps the reasons for great mismatching between GTV_MRI_ and GTV_PET_ are as follows. Owing to its poor spatial resolution and partial volume effect, PET may be inferior to T_2_W‐MRI for anatomic visualization of the esophageal wall.^28^ Furthermore, the SUV value of PET‐CT selected in delineating GTV may be so low as to including normal periesophageal tissue, which would reduce the accurate in target delineation inevitably. Finally, it should be noted that, the inconsistency of breathing pattern during the acquisition of T_2_W‐MRI and PET‐CT might affect the position and shape of the tumor to some extent.^8^, ^12^ As mentioned earlier, the size of GTV derived from MRI was significantly less than that from PET‐CT. In conclusion, GTV_PET_ may include more information of respiration motion than GTV_MRI_. GTV_MRI_ could not replace GTV_PET_ due to the variation in target volume information included in each imaging. What's more, there would be a target dismiss whether GTV_MRI_ or GTV_PET_ was regarded as therapeutic target area independently.

It is crucial to distinguish the upper and lower margins of the GTV for radiotherapy of esophageal carcinoma. The longitudinal length of GTVs delineated on 3DCT or 4DCT is often overestimated.^29^ Currently, esophageal X‐ray, endoscopy, and endoscopic ultrasonography with auxiliary metal clip marking are recommended to determine the longitudinal length of GTVs, which were thought to be superior to CT scan. However, the above images remained different imitations in themselves.^29^, ^30^ Although, PET‐CT has showed certain value in measuring the longitude in the length of esophageal carcinoma, its clinical application has always been challenged because the accuracy of PET‐CT is easily affected by many factors such as inflammation, SUV value et al^31^ With advances toward MRI‐guided imaging technique, combining DW‐MRI with CT images is regarded as a reliable tool for tumor length determination.^32^ As expected, our research showed that the tumor length measured by GTV_PET2.5_or GTV_DWI_ images was similar to the length measured by endoscopy, and there was no significant difference between the longitudinal length derived from GTV_PET2.5_ and GTV_DWI._. Conclusively, DW‐MRI could be used to replace PET‐CT for determining the upper and lower boundaries of esophageal carcinoma.

## 5 CONCLUSIONS

The GTV size of primary esophageal carcinoma derived from T_2_W‐MRI is similar to that from EE phase of 4DCT, but the similarity and inclusion relation between the GTVs form the two images were unsatisfied. The MRI imaging could not include entire respiration. It may be a good choice to guide target delineation and construction of esophageal carcinoma by combining 4DCT with MRI imaging. There are significant differences for the GTVs in size and spatial position derived from T_2_W‐MRI and ^18^F‐FDGPET/CT. Further research is needed to determine the necessity of combining PET‐CT and MRI in target delineation. Whereas, utilization of DWI in treatment planning for esophageal cancer may provide further information to assist with target delineation. Further studies are needed to determine if this technology will translate into meaningful differences in clinical outcome.

## AUTHORS' CONTRIBUTIONS

Authors' contributions HML contributed to the study design, the patient enrollment, the data statistics and analysis and writing the manuscript. FXL and JBL participated in the study design. YZZ, YJZ, and YLG contributed to reviewing the delineation. MX, QS, and XJL made important contributions in collecting the data and revising the content. All authors read and approved the final manuscript.

## ETHICS APPROVAL AND CONSENT TO PARTICIPATE

Ethics approval and consent to participate Approval was obtained from the institutional research ethics board of the Shandong Tumor Hospital Ethics Committee.

## Data Availability

The datasets used and/or analyzed during the current study are available from the corresponding author on reasonable request.
