# Erratum

**DOI:** 10.1002/cam4.3521

**Published:** 2020-10-02

In the article by Li et al.,^1^ the funding program number or grant number for National Natural Science Foundation Item is incorrect. The correct funding program number is 81773287, instead of 1773287.
